# Enhancement of plant growth regulators production from microalgae cultivated in treated sewage wastewater (TSW)

**DOI:** 10.1186/s12870-022-03764-w

**Published:** 2022-07-29

**Authors:** Walaa M. Elakbawy, Sanaa M. M. Shanab, Emad A. Shalaby

**Affiliations:** 1 Department of Botany and Microbiology, Faculty of Science, Cairo University, Giza, 12613 Egypt; 2 Biochemistry Department, Faculty of Agriculture, Cairo University, Giza, 12613 Egypt

**Keywords:** Bioassays, Callus, Chlorophyta, Cyanobacteria, Sewage wastewater

## Abstract

The aim of this work is to develop an efficient method for detection and evaluation of the plant growth regulators produced from cyanobacteria species (*Anabaena oryzae* and *Nostoc muscorum*) cultivated on BG11_0_, and Chlorophyta alga (*Chlorella vulgaris*) cultivated on BG11 in addition to the cultivation of these strains on treated sewage wastewater (TSW) combined with control media (BG11 and BG11_0_) at different concentrations (100, 75 and 50%). Bioassays were performed on Wheat coleoptile length and Cucumber cotyledons fresh weight for indole acetic acid (IAA) and Benzyl adenine (BA) detection. In addition, application experiments of IAA and BA presence in algal extract were applied on tomato plantlets and soybean callus. The obtained results of *A. oryzae* and *N. muscorum* extracts (grown on BG11_0_ and 100% sewage media) with optimum conc. of IAA and BA showed moderate shoot length and leaves number as well as high root initiation of tomato explant compared to control. While dimethyl sulfoxide (DMSO), IAA conc. as well as IAA + BA conc. showed no effect on branching and leaf expansion. The results of *C. vulgaris (grown* on BG11) also revealed that the shoot had high leaves number and greatest root initiation, without branching and leaf expansion. On the other hand, 100% TSW had a moderate shoot, leaves number and high root initiation. Extracts of *A. oryzae* and *N. muscorum* (grown on BG11_0_) induced 1.5-fold increase in soybean callus fresh weight, while the growth on 100% TSW was shown to be less effective. Moreover, extract of *C. vulgaris* (grown on BG11) induced a moderate effect, while its growth on 100% TSW was shown to be less effective in soybean callus fresh weight increment.

## Introduction

Algal cultivation on wastewater could be potentially useful to reduce or even eliminate nutrients and heavy metals in the wastes. This system could use the wastewater instead of being pumped into the ocean and contaminate its water every day. The harvested algal biomass has many potential uses, which include bio-fuel, fish feed and ethanol production. The algae can help the elimination of harmful chemicals out of the wastewater and produce clean drinking water. Microalgal cultures on treated wastewater (urban, industrial or agricultural effluents) can provide a tertiary biotreatment coupled with the production of potentially valuable algal biomass, which can be involved or useful for various purposes [1].

Several bioactive compounds are produced by cyanobacteria and microalgae have been discovered by screening programs [2]. Many of these chemicals have a diverse range of biological activities and chemical structures, which affect many physiological and biochemical processes within the cell. Such chemicals are thought to be related to the regulation and succession of algal and bacterial populations. These chemicals are expected to be synthesized under stress conditions, low algal growth rate and released at appropriate concentration to be effective.

In 1960-1970s an active search for phytohormones in various algal taxa was performed. During this period many compounds with hormonal activity were detected from Chlorophyta, Phaeophyta and Rhodophyta. The most interesting of them are IAA, GA_3_, IPA and lunularic acid. Algae produce plant growth regulators [PGRs], similar to those of higher plants. Cyanobacteria synthesized various amounts of auxin in the presence of different concentrations of its precursor L-Tryptophan [3].

Many studies focused on the importance of micro-and macro-algal filtrates and extracts either added to the soil or mixed with the tissue culture media or even applied as foliar spray. The effect of seaweed extracts made from the brown algae *Durvillaea potatorum* and *Ascophyllum nodosum* on tomato plant and soil was investigated [4–6]. They showed a pronounced beneficial effect not only on soil but it can also enhance the morphological, physiological and biochemical parameters of different crop plants and vegetables [7–9]. Seaweed extracts contained growth promoting substances as phytohormones and other bio-stimulants like amino acids, vitamins, macro-and micronutrients. These biostimulants may induced an enhancement effect to plant growth [10] by facilitating water uptake, root and shoot growth and tolerance to stresses on all plant developmental stages (as germination of seeds, growth of plants seedlings till plant harvest). Lower concentrations were found to be more effective either applied for seed germination or seedling growth [11, 12].

Plant growth regulators are found to play a very important role in plant tissue culture (PTC). They are vital for many growth phases. Also, they help in studying the morphological, biochemical, cytological and genetical events of the plants [13]. Auxins and cytokinins are the most widely applied PGRs in plant tissue culture and they are usually added simultaneously. The ratio of auxin to cytokinin control the type of culture to which the explant will develop. Application of higher auxin concentrations stimulates root formation, whereas higher cytokinin regenerates shoot while, intermediate ratio favors callus production. All these events depend on the internal amounts of hormones inside the explants. Since its discovery, PTC had significant and valuable impacts in agricultural sciences and constitutes an essential tool in the advancement of modern agriculture [14].

This research aimed to detect and evaluate the plant growth regulators produced from some microalgal species cultivated on low quality water (secondary treated sewage wastewater) and confirm their presence and activity using bioassays and tissue culture application techniques.

## Materials and methods

### Algal samples

#### Algal species and culture conditions

Two cyanobacterial species (*Anabaena oryzae* and *Nostoc muscorum*) were obtained from the Microbiology Department, Soils, Water and Environment Res. Inst. (SWERI), Agric. Res., Center (ARC) and one green alga species (*Chlorella vulgaris.*) was provided from Dr. Sanaa M. Shanab culture collection, Department of Botany and Microbiology, Faculty of Science, Cairo University, 12,613 Giza, Egypt.

The cyanobacteria species were cultured and maintained on liquid BG11_0_ free from nitrogen [15] for cyanobacteria and BG11 media [16] for the green alga. Cultures were incubated in the incubator under continuous aeration (1.25 l/min), 16: 8 h light and dark cycle and light intensity of 40 µE/m^2^/s at 25 ± 1 °C for 30 days.

### Wastewater sources

Sewage Wastewater (SWW, Urban) was obtained from Zenain wastewater station, Al-Eshreen, Giza, Egypt.

### Wastewater analysis

Chemical indicators and parameters of treated wastewater and BG11 media were analyzed according to APHA [17] as shown in Table 1.

**Table 1:** Chemical characteristics of the treated sewage wastewater (TSW) used for cultivation of microalgae compared with the composition of BG11 medium

Parameters	BG11 medium	Treated sewage wastewater (TSW)
pH	7	6.48
Total nitrogen mg/L	248	28.5
Cation’s (mg/L)		
Ca^2+^	9.8	36.7
Mg^2+^	8.8	16.2
K^+^	17.9	17.5
Na^+^	140	177
Anion’s (mg/L)		
CO_3_^2−^	6.2	39.9
HCO^3−^	13	183
CI^−^	18	71
SO_4_^2−^	37	263
Trace elements (μg/L)		
Cu	20	36
Zn	5	62
B	500	150
P	7000	6100
Fe	3200	130
Co	16	27
Mn	500	110

### Treatment of sewage wastewater

The SWW (sewage wastewater) was sterilized by microfiber filter (0.22 µm) to get rid of large particles and bacteria. The wastewater was then termed treated sewage wastewater (TSW). It was applied separately (as 100% TSW) and in combination with BG11_0_ or BG11 (in 75, 50%) to study their impacts on algal strain**s** [18]. The BG11_0_ or BG11 media were used as control media the (standard synthetic media). The algal strains were grown in 500 ml Erlenmeyer flasks to which 10% algal inocula were applied. Each experiment was conducted in triplicates and cultures were incubated in an illuminated incubator with the previously mentioned conditions.

### PGRs Extraction

The harvested algal cells were dried to powder and extracted by 96% methanol according to the method described in El Akabawy et al*.* [19].

The algal cells were harvested in each experiment at the end of the exponential phase, centrifuged, oven dried (50 °C for 24 h), and extracted overnight in 96% methanol. Then, the methanolic fraction was filtrated, and the residual pellets were re-extracted 3 times with 40% (10 ml) cold methanol. The combined methanol extracts were evaporated in the dark at room temperature. The residual aqueous solution was adjusted to pH 2.6 residual aqueous solutiontimes by absolute ethyl acetate (50 ml/each extract). In addition, the ethyl acetate fraction was separated and dried over anhydrous MgSO4. Finally, the residue was dissolved in 4 ml of absolute methanol.

### Experimental research and field studies on plants

All Experimental research and field studies on plants, including the collection of plant material (including seeds), comply with relevant institutional, national, and international guidelines and legislation.

### Response of the wheat coleoptile section elongation to auxins

This method was applied according to Nitsh and Nitsh [20], where: Wheat grains (*Triticum aestivum L*. Giza 171 was obtained from Agriculture Research Center (ARC), Giza, Egypt) were soaked in distilled water for two hours. The soaked grains were grown in darkness at 25 C for 48 h under green–safe light, then, 6 mm sections below the tips were cutten and10 coleoptile sections of 6 mm length were incubated (in darkness at 25 C for 24 h.) in 2.5 ml sucrose buffer pH 5 with 2.5 ml of algal crude extract of tested samples and 2.5 ml of different IAA conc. (10^–8^-10^−3^ M) for dose response curve. Elongation of wheat coleoptile was determined in mm.

### Cucumber cotyledon fresh weight for cytokinin’s bioassay

Cucumber seeds (*Cucumis sativus L.* picoline F1-hybrid) were purchased from Germany supermarket) and was kindly identified in the Department of Botany and Microbiology, Faculty of Science, Cairo University. Seeds were germinated in darkness for 4 days at room temperature (25 °C) on moist filter paper in 5-cm petri dishes. Cotyledons were excised excluding petiole tissues and four cotyledons were placed in each petri-dishes after determining their fresh weights. The cotyledons were placed with their ad axial sides down on the paper. They were incubated in the incubator at 25 ± 2 °C and 12/12 h light–dark photoperiods. Three mL of crude extracts of each tested alga was applied to each petri dish at BA concentrations 5, 10, 25, 50 and 100 ppm which were used for dose response curve. Cotyledon growth was measured by determining the increase in fresh weights (in grams) as reported by Letham [21].

### HPLC conditions

The standard hormonal samples (were purchased from Sigma-Aldrich) and the algal methanol extracts were analyzed by high performance liquid chromatography (HPLC) with the same conditions published in El Akbawy et al*.* [19]. The amount of IAA and BA in the algae grown on BG11_0_ or BG11 (alone and in combinations) with TSW were estimated from the dose growth curves and the HPLC analysis of algal hormones.

### Application experiments

*A. oryzae, N. muscorum* and *C. vulgaris* grown on different media were selected for application experiments to confirm the presence of IAA and BA by HPLC analyses of algal extracts.

### Soybean callus and Tomato plantlets for Benzyl adenine (BA) and Indol aceticacid (IAA) detection

The applied methods were developed by Miller [22] and Ran et al*.* [23].

#### Seed sterilization

The seeds of soybean (*Glycine max* L. cv. Giza 111) was obtained from Agriculture.

Research Center (NRC), Giza, Egypt) and tomato (*Lycopersicon esculentus* L. cv. moneymaker) was purchased from USA). The plant seeds were kindly identified in the Department of Botany and Microbiology, Faculty of Science, Cairo University. Seeds sterilization was performed according to the method of Elakbawy et al. [19].

#### Seed’s germination

All seeds (soybean and tomato) were germinated separately in vitro on basal Murashige and Skoog medium (MS) [24]. To which was added 30 g/L sucrose and 100 mg/L myo-inositol and solidified with 0.8% agar. All jars were incubated in growth room with constant temperature (25 ± 0.2ºC) and photoperiod (16 h dark / 8 h light) for7 days.

#### Seedling of soybean

Aseptically growing soybean seedlings of 7 day-old were used as a source of cotyledon explants for callus induction.

#### Soybean callus induction

Soybean callus cultures of were initiated by placing the excised sterile cotyledon-explants (2–3 mm) thickness of 7 day-old. The aseptically grown seedlings were placed in 200 ml screw-capped glass jars containing 25 ml callus induction media. Callus induction medium contained MS supplemented with 0.5 mg/L BA, 30 g/L sucrose, 100 mg/L myo-inositol and 0.8% agar. Five explants per jar (triplicates) were cultured. All cultures were cultured in the dark at constant temperature (25 ± 2 C) for 2 weeks.

### Experiment on soybean callus

Three pieces of soybean calli were transferred to each jar containing 25 ml of basal MS medium solidified with 0.8% agar as control. Treatments were carried out using different concentration of BA (10, 25 and 50 ppm), crude extract of the algae grown on BG-11_0,_ BG11 and TSW. Each treatment was represented by 3 jars (as triplicates). The jars were maintained for 28 days at 25 ± 0.2ºC in darkness. Data were determined as fresh mass of calli per jar.

### Seedling of tomato

Aseptically grown tomato seedlings (14 day-old) were applied as a source of plantlets.

### Experiment of tomato seedlings

Apical buds of tomato seedlings were excised and transferred to MS media with different additives as described in Elakbawy et al*.* [19].

### Statistical Analysis

Data were subjected to an analysis of variance, and the means were compared using 214 the Least Significant Difference (LSD) test at the 0.05 levels (*p* ≤ 0.05), followed the method described by Snedecor and Cochran [25].

## Results and discussion

### Growth rate with different treatments

The growth rate was expressed as dried mass (mg per 10 ml sample) then evaluated as dry weight (gm/L) and optical density (OD) at 550 nm for cyanobacteria and 660 nm for Chlorophyta. The samples were collected along 30 days with 5 days interval.

**Fig 1:**
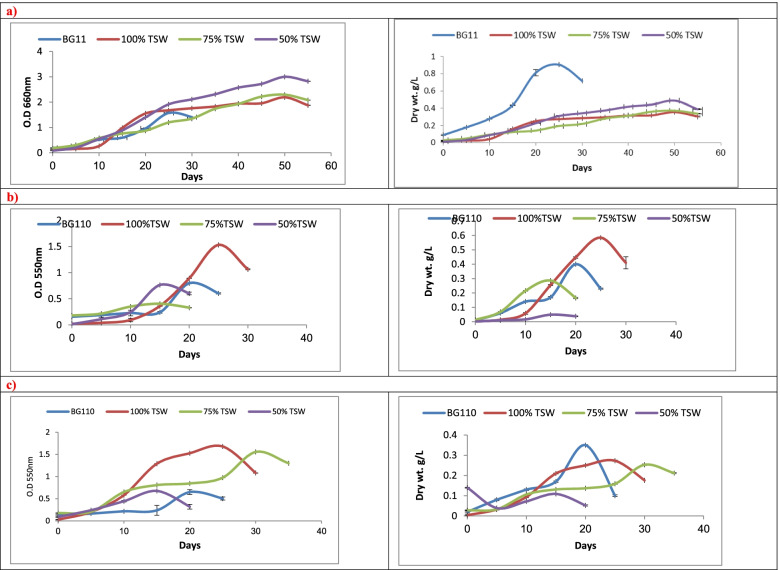
Growth rate by O.D550nm, O.D660nm and Dry wt. ( as g/L) of **a**) *Chlorella vulgaris* **b**) *Anabaena oryzae* **c**) *Nostoc muscorum* cultivated on different conc. of treated sewage wastewater (TSW) in combination with BG11 and BG110

Figure 1A showed the growth rates of all *Chlorella vulgaris* treatments as with the compared control ones. It illustrated that the growth rates have short lag phases (at day 5), then various length of log phases in addition to short decline phases. In case of control medium (BG11 and TSW treatments), the growth rates expressed gradual increment starting from day 1 of cultivation until they reached the optimum biomass productivity then the growth declined. The day of the maximum productivity differ from treatment to another. *C. vulgaris* treated with control medium BG11 reached maximum growth rates (900 mg/L) at day 25 of cultivation, which is twice the value attained with 50% TSW at day 50 of cultivation. In addition, treating *C. vulgaris* with control medium (BG11) expressed absolutely high productivity over other treatments even in the decline phase (715 mg/L) at day 30 of cultivation. However, *C. vulgaris* demonstrated lower growth rates recording 356 and 373 mg/L, respectively at day 50 of cultivation, when treated with 100% and 75% TSW as shown in Table 2. Similarly, optical density method expressed the same trend of *C. vulgaris* treated with control medium BG11 and the productivity stills high compared to the values of other treatments.

**Table 2:** Maximum biomass productivity (as mg/L) of algal species cultivated on control media (BG 11 and BG11_0_) and treated sewage wastewater (TSW)

Algal species	Maximum biomass productivity by BG11 and BG11_0_	TSW treatment	Maximum biomass productivity by TSW
***A. oryzae***	400 mg/L (BG11_0_)	100% TSW	600 mg/L
		75% TSW	300 mg/L
		50% TSW	50 mg/L
***N. muscorum***	350 mg/L (BG11_0_)	100% TSW	273 mg/L
		75% TSW	253 mg/L
		50% TSW	109 mg/L
***C. vulgaris***	900 mg/L (BG11)	100% TSW	356 mg/L
		75% TSW	373 mg/L
		50% TSW	450 mg/L

*TSW* Treated sewage wastewater

The growth rates of all *Anabaena oryzae* treatments and control medium (BG11_0_) appeared with 3 phases (short lag phase, log phase and short decline phase), as shown in Fig. 1B. It was clearly noticed that the growth rate of control medium (BG11_0_) and treatments rise continuously from the first cultivation day until they get the maximum biomass productivity (differ according to treatments) then growth was diminished. Optimum growth rate of *A. oryzae* cultured on control medium (at day 20 of cultivation) represented about 0.67 of the value recorded at day 25 of cultivation when treated with 100% TSW (600 mg/L). Also, treating *A. oryzae* with 100% TSW (at day 30) and control medium (at day 25) expressed maximum productivity (400 mg/L) compared to those of other treatments as shown in Table 2. However, low growth rates expressed 50 and 300 mg/L at day 15 of cultivation, when treated with 50% and 75% TSW, respectively which was lower than the optimum value recorded by control medium.

The growth rates of all *Nostoc muscorum* treatments and control BG11_0_ were manifested in Fig. 1C. The growth rate of control medium (BG11_0_) and treatments showed similar trends but vary with the days of lag, maximum productivity and decline. Maximum productivity of *N. muscorum* as shown in Table 2 reached about 350 mg/L at day 20 of cultivation when treated with control medium (BG11_0_). However, treatment with 50% TSW expressed lowest growth rate recording 109 mg/L (at day 15 of cultivation), which was about 1/3 the value recorded with control medium (350 mg/L) at day 20 of growth. Treating *N. muscorum* with 75% TSW and 100%TSW represented about 72 (at day 30) and 78% (at day 25), respectively of the optimum productivity of that expressed with control medium.

The results revealed that maximum biomass productivity was enhanced using 100% TSW in case of cyanobacterial species (*A. oryza* & *N. muscorum*) and using 50% TSW for green *C. vulgaris* than the control one (BG11 or BG11_0_). These may be due to the richness of TSW with macro and micro-elements in addition to IAA, BA and NPK compared to the utilized synthetic media (BG11 and BG11_0_).

The results were in context with those reported by Wang et al*.*, [18] who mentioned that, some microalgae (as *C. vulgaris*) could effectively acclimatize on various wastewaters (municipal wastewater treatment plant, MWTP) without lag phase. Similarly, Shalaby et al., [26] described that *N. muscorum* was adapted well when cultivated on several types of wastewaters (Sewage, Industrial and Agriculture).

Table 1 recorded the chemical analysis of both TSW and BG11_0_ media which overlook the reason that TSW stimulated the higher growth rates of algae. TSW was slightly acidic while BG11_0_ medium was neutral. Both TSW and BG11_0_ media have equal content of potassium. The total nitrogen amount was approving in TSW medium than of those of BG11_0_. Also, TSW possessed high amounts of anions and trace elements (Cu, Zn and Co) necessary in enzymes and biochemical processes. While, the amounts of boron, phosphate, iron and manganese were higher in BG11_0_ medium.

### Dose response curve of IAA

Table 3 recorded the elongation of wheat coleoptile sections after treatment by various IAA concentrations (10^–8^- 10^–3^ M). The results revealed that the increase in exogenous IAA concentrations caused a gradual enhancement in wheat coleoptile sections elongation up to the conc. 10^–5^ M IAA where maximum elongation was recorded (8.9 mm, 70.6%) and compared to that obtained by control (7.7 mm). Elevation of IAA conc. (10^–4^, 10^–3^ M) showed an inhibitory effect

**Table 3:** Dose response curve of Indole acetic acid (IAA) using wheat coleoptile section elongation (in mm) All data

concentration of IAA (M)	Initial coleoptile length (mm)	Mean of final coleoptile length (mm)	Mean of net coleoptile elongation (mm)	Change from control (mm)	Percentage (%)
Control	6	7.7	1.7	-	-
10–^8^	6	8.3	2.3	0.6	35.3
10–^7^	6	8.4	2.4	0.7	41.2
10–^6^	6	8.8	2.8	1.1	64.7
10–^5^	6	8.9	2.9	1.2	70.6
10–^4^	6	7.6	1.6	-0.1	-5.9
10–^3^	6	6.8	0.8	-0.9	-52.9
*Chlorella vulgaris* ext. on BG11 (0.017 ppm)	6	7.8	1.5	-0.2	-11.8
*Anabaena oryzae* ext. on BG110 (0.24 ppm)	6	7.92	1.92	0.22	12.9
*Nostoc muscorum* ext. on BG110 (0.6 ppm)	6	7.6	1.6	-0.1	-5.9

All data represent the mean values of 10 replica, initial length = 6 mm

The replacement of the exogenous IAA concentration by crude algal extract of *A. oryzae* cultivated on BG11_0_ shown an appreciable positive result with more elongation values than that produced by 10^–4^ M.

IAA (7.92, 12.9%), while crude extracts of both *Chlorella vulgaris* (cultivated on BG11) and *Nostoc muscurum* grown on BG11_0_ shown negative result with elongation values which were approximately equal to that produced by 10^–4^ M IAA (7.5, -11.8% and 7.6, -5.9%) as recorded in Table 3.

The induced elongation of wheat coleoptile sections (after the removal of tip rich in auxin), by applying exogenous IAA, could be explained as following: The plant cell wall is rigid and not easy to expand due to its composition of cellulose microfibrils mixed with polysaccharides and embedded in hemicellulose, pectin, proteins and other components which are held together by hydrogen bonding. So the expansion of cell wall need the change of its physical properties, where disruption of the cellulose microfibrils structure and loosening the link with polysaccharides takes place by auxins especially at its optimum conc. Auxins promote acidification of the cell wall(due to acid growth theory), stimulating the plasma membrane in the cytoplasm to pump protons (H^+^) to the cell wall which induce loosening enzymes to loosen the cross links between the cellulose microfibrils, polysaccharides and the hydrogen bonding and so microfibrils displace to slide past each other allowing the cell wall to increase its extensibility and plasticity to expand. Then the bonds are reformed in the new position and microfibrils stimulated inducing irreversible cell wall growth [27].

### Dose response curve of BA

Table 4 recorded the growth curve of the exogenously added BA to cucumber cotyledons using concentrations 5- 100 ppm.

**Table 4:** Dose response curve of Benzyl adenine (BA) using fresh wt. (gm) of cucumber cotyledon

Concentration of BA [ppm]	Fresh wt. of cucumber cotyledon (gm)
control	0.0127
5	0.06
10	0.0826
25	0.123
50	0.088
100	0.086
*Chlorella vulgaris* ext. on BG11 (0.017 ppm)	0.0002
*Anabaena oryzae ext.* on BG110 (0.24 ppm)	0.006
*Nostoc muscorum ext.* on BG110 (0.6 ppm)	0.0072

All data represent the mean values of triplicates

The obtained data revealed that, lower concentration of BA (5 and 10 ppm) induced gradual increase in cotyledon fresh weight which reached its maximum weight (0.123 g) at the optimum BA conc. (25 ppm) compared to that of the control plant (0.127 g).

Elevation of BA conc. (50 and 100 ppm) caused a steady constant inhibition of growth (≈0.08 g) compared to those recorded by lower BA conc. (5 and10 ppm).

The replacement of exogenous BA conc. by crude algal extract of *A. oryzae* (cultivated on BG11_0_) was shown to produce 0.006 g of cucumber cotyledon fresh weight with 0.24 ppm. Whereas crud algal extract of *N. muscorum cultivated* on BG11_0_ was shown to produce 0.0072 g of cucumber cotyledon fresh weight induced by 0.6 ppm and *C. vulgaris* crud extract (cultivated on BG11) produced 0.0002 g with 0.017 ppm.

The optimum conc. of IAA and BA in Tables 3 and 4 were used to calculate the hormone conc. in the application experiments using wastewater, BG11 and BG11_o_ media as well as IAA + BA combinations.

### HPLC analysis results

Analysis of plant growth regulators IAA and BA in different extracts of the tested algae cultivated in TSW (100, 75 and 50%), BG11 and BG11_0_ media were performed using HPLC as shown in Table 5. The analyses showed that, IAA content in *Chlorella vulgaris* was increased in alga cultivated in 50 and 100% TSW and BG11 combination (0.014 and 0.032 mg/g) when compared with only BG11 medium (0.01 mg/g). While, the other results showed decreased values compared with BG11 medium. BA amount was increased in 100, 75 and 50% TSW and BG11 combination (0.695, 0.005 and 0.033 mg/g). While, TSW 100% recorded the highest concentration of IAA and BA when compared with values obtained by BG11 medium.

**Table 5:** HPLC analysis of PGRs (as mg/g) extracted from cyanobacteria species and Chlorophyta alga cultivated on BG11_0_ or BG11 (as a control) and TSW media

Algal extract	Hormone	R_t_ (min)	100% TSW		75%TSW		50%TSW		Control medium	
			Conc. mg/g	R%	Conc. mg/g	R%	Conc. mg/g	R%	Conc. mg/g	R%
*A. oryzae*	IAA	9.4	0.051	67.5	0.012	38	0.011	17.7	0.003	66.7
	BA	10.5	0.023	32.5	0.021	62	0.044	82.3	0.013	33.3
*N. muscorum*	IAA	9.4	0.053	31.5	0.025	88.6	0.050	67.5	0.002	14.49
	BA	10.5	0.120	68.5	0.004	11.4	0.017	32.5	0.014	85.51
*C. vulgaris*	IAA	9.4	0.032	4.4	0.005	70.2	0.014	28.5	0.01	75.3
	BA	10.5	0.695	95.7	0.005	29.8	0.033	71.5	0.004	24.7

*TSW* Treated sewage wastewater, *IAA* Indole acetic acid, *BA* Benzyl adenine, *R%* Relative percentage

The obtained results of different *Anabaena oryzae* extracts cultivated in TSW and BG11_0_ media revealed that, both IAA and BA contents were increased in alga cultivated in BG11_0_ and TSW combination media (50, 75 and 100%), when compared with control (BG11_0_ medium). Moreover, 100% TSW recorded highest concentration of IAA (0.051 mg/g) when compared with its content when grown on BG11_0_ medium (0.003 mg/g). However, algae cultivated in 50% TSW recorded the highest concentration of BA (0.044 mg/g) when compared with those recorded on using BG11_0_ medium (0.013 mg/g).

The analysis of various extracts of *N. muscorum* showed that, IAA content was elevated in alga cultivated in TSW (50, 75 and 100%), BG11_0_ combination over that of control. The amount of BA was improved in 100%, 50% TSW, BG11_0_ combination media and declined in 75% TSW. However, the highest concentration of IAA and BA was recorded in 100%TSW when compared with BG11_0_ medium.

These results showed that the abiotic stress performed by the investigated algae with treated wastewater (rich in macro and micro nutrients as seen in Table 1, could stimulate algal cells to induce various secondary metabolites as self-defense. These obtained results were in accordance with those recorded by Rodríguez-Meizoso et al*.* [28] who mentioned that when Microalgae face stress and/or extreme natural environmental conditions, they rapidly acclimatize themselves to the new conditions for survival. In this process, they induce new biologically active secondary metabolites which are not synthesized under normal unstressed conditions.

### Tomato experiment

This *invitro* experiment tested the influence of different auxin (IAA) and cytokinin (BA) concentrations (separately and in combination), in addition to the tested algal optimum conc of these hormones and its crude extracts obtained from cultures grown on various tested media (synthetic and cheap media). MS and DMSO media was used as controls. The impact was focused on the morphological parameters (shoot length, leaves numbers, leaf expansion, roots initiation and branching) of tomato plantlets.

**Fig 2:**
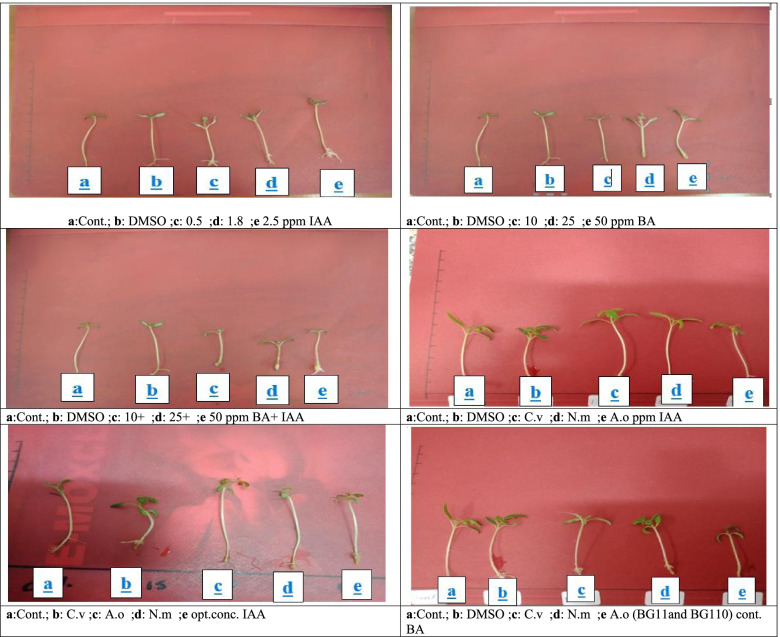
Growth of tomato explants by syn. BA, IAA and mixtures of IAA + BA conc. as well as algal extracts (grown on various media) with optimum conc. of BA and IAA (from dose response curve) on tomato plant

Table 6 and Fig. 2 revealed that *A. oryzae* which showed that, IAA at conc. 1.8 ppm stimulated the greatest shoot length (6.850 ± 1.588) compared to MS and DMSO media, other IAA conc. (0.5 and 2.5 ppm) and IAA + BA conc. This unbranched shoot possessed high leaves number (3.583 ± 0.515) and greatest root initiation. High IAA conc. (2.5 ppm) was highly effective on leaves number and shoot length than the lower IAA conc. (0.5 ppm).

**Table 6:** The effect of *Anabaena oryzae* crude extracts (Grown on BG11_0_ and 100% TSW) with optimum conc. of acetic acid (IAA) and Benzyl adenine (BA) (obtained from dose response curve) on Tomato plant

Parameter	Shoot Length (cm)	Leaves, numbers	Root initiation	Branching	Leaf expansion
Control (MS medium)	5.217 ± 0.469^bcd^	3.500 ± 0.674^a^	+ + +	-	-
DMSO	5.083 ± 0.925^ cd^	3.417 ± 0.669^a^	+ + +	-	-
IAA ( 0.5 ppm)	4.850 ± 0.454^cde^	3.083 ± 0.900^ab^	+ + + + + +	-	-
IAA (1.8 ppm)	6.850 ± 1.588^a^	3.583 ± 0.515 ^a^	+ + + + + +	-	-
IAA (2.5 ppm)	6.008 ± 0.734 ^a b^	3.750 ± 0.452 ^a^	+ + +	-	-
BA ( 10 ppm)	4.533 ± 0.624^bc^	2.833 ± 0.577 ^bc^	+	-	+
BA (25 ppm)	4.442 ± 0.516^b c d^	3.500 ± 0.674 ^ab^	+	+ +	+
BA (50 ppm)	4.425 ± 0.636^b c d^	3.333 ± 0.651^a b^	-	+ + +	+ + +
IAA + BA (0.5 + 10) ppm	4.008 ± 0.478 ^e^	3.167 ± 0.577^ab^	+ + +	-	-
IAA + BA (1.8 + 25) ppm	4.533 ± 0.414 ^d e^	3.667 ± 0.492 ^a^	+ + + +	+	-
IAA + BA (2.5 + 50) ppm	4.525 ± 0.398 ^d e^	2.500 ± 0.674 ^b^	+	-	-
*A. oryzae* IAA optim. Conc. ( BG-11_0_)	5.208 ± 0.348^bcd^	2.583 ± 0.515 ^b^	+ + +	-	-
*A. oryzae* BA optim conc.(BG11_0_)	4.8833 ± 0.3433^b^	2.500 ± 0.522^c^	+ +	-	-
*A. oryzae*(100% TSW) IAA optim. conc	5.708 ± 1.053^bc^	3.750 ± 0.452 ^a^	+ + + + +	-	-
*A. oryzae*(100%TSW) BA optim. conc	4.767 ± 0.543^b^	3.083 ± 0.669^abc^	+ + +	-	-

*TSW* Treated sewage wastewater (-):Absence (6 +): the highest (5 +):high (4 +):moderate (3 +):low ( +):The lowest DMSO: Dimethyl sulfoxide MS: Murashige and Skoog medium

The values are the mean of three replicates ± SD

Values followed by different letters are significantly different (*p* ≤ 0.05)

Moderate conc. of exogenously applied hormones (IAA + BA, 1.8 + 25 ppm) enhanced the morphological parameters of tomato explants. Similar results were observed by *A. oryzae* extract cultivated on BG11_0_ supplied with 0.5 IAA + 10 BA ppm. However, higher IAA + BA mixture conc. (2.5 + 50 ppm) expressed lesser stimulatory effects to all morphylogical parameters. *A. oryzae* extracts (cultured on BG11_0_ and 100% TSW media), has no effect on branching and leaf expansion but stimulated high root initiation with moderate shoot length and leaves number compared to control, DMSO, IAA conc. and IAA + BA concentration. Table 6 and Fig. 2 revealed that the crude extract of *A. oryzae* with optimum conc. of BA expressed moderate shoot length, leaves number and root initiation.

Table 7 and Fig. 2 presented that, indole acetic acid (1.8 ppm) exhibited the greatest shoot length (6.850 ± 1.588), leaves number (3.583 ± 0.515) and root initiation compared to control media, other IAA conc. (0.5 and 2.5 ppm) and the combination IAA + BA concentration. *N. muscorum* extract (grown on BG11_0_ and 100% TSW media) had moderate shoot length and high leaves number of tomato explant Highest root initiation were recorded by utilizing 100% TSW and moderate was observed by BG11_0_ medium. The crude extract of *N. muscorum* supplemented with optimum conc. of BA (grown on BG11_0_ and 100% TSW) stimulated moderate shoot length (4.892 ± 0.470, 4.833 ± 0.597) as well as low root initiation and leaves number (3.250 ± 0.754, 3.083 ± 0.669), Table 7 and Fig. 2.

**Table 7:** The effect of ***Nostoc muscorum*** crude extracts (Grown on BG11_0_ and 100% TSW) with optimum conc. of acetic acid (IAA) and benzyl adenine (BA) (obtained from dose response curve) on Tomato plant

Parameter	Shoot Length (cm)	Leaves, numbers	Root initiation	Branching	Leaf expansion
Control (MS medium)	5.217 ± 0.469^bcd^	3.500 ± 0.674 ^a^	+ + +	-	-
DMSO	5.083 ± 0.925^ cd^	3.417 ± 0.669 ^a^	+ + +	-	-
IAA ( 0.5 ppm)	4.850 ± 0.454^cde^	3.083 ± 0.900^ab^	+ + + + + +	-	-
IAA (1.8 ppm)	6.850 ± 1.588 ^a^	3.583 ± 0.515^a^	+ + + + + +	-	-
IAA (2.5 ppm)	6.008 ± 0.734 ^a b^	3.750 ± 0.452 ^a^	+ + +	-	-
BA ( 10 ppm)	4.533 ± 0.624^bc^	2.833 ± 0.577^bc^	+	-	-
BA (25 ppm)	4.442 ± 0.516^bc^	3.500 ± 0.674^ab^	+	+ +	+
BA (50 ppm)	4.425 ± 0.636^bc^	3.333 ± 0.651^ab^	-	+ + +	+ + +
IAA + BA (0.5 + 10) ppm	4.008 ± 0.478 ^e^	3.167 ± 0.577^ab^	+ + +	-	-
IAA + BA (1.8 + 25) ppm	4.525 ± 0.414 ^de^	3.667 ± 0.492 ^a^	+ + + +	-	-
IAA + BA (2.5 + 50) ppm	4.525 ± 0.398 ^d e^	2.500 ± 0.674 ^b^	+	-	-
*N. muscorum* IAA optim conc.(BG-11_0_)	5.367 ± 0.350^bcd^	3.333 ± 0.651^a^	+ + +	-	-
*N. muscorum* BA optim conc. (BG-11_0_)			+ + +	-	-
*N.muscorum* (100%TSW) IAA optim. conc	5.692 ± 0.392^bc^	3.750 ± 0.452^a^	+ + + + + +	-	-
*N. muscorum*(100%TSW) BA Optim. conc			+ +	-	-

*TSW* Treated sewage wastewater (-): Absence (6 +): the highest (5 +):high (4 +):moderate (3 +):low ( +):The lowest DMSO: Dimethyl sulfoxide MS: Murashige and Skoog medium

The values are the mean of three replicates ± SD

Values followed by different letters are significantly different (*p* ≤ 0.05)

The results of *C. vulgaris* as shown in Table 8 and Fig. 2 revealed that 1.8 ppm indole acetic acid (IAA) reported the formation of greatest shoot length (6.850 ± 1.588, 6.817 ± 0.746) compared to other treatments. The shoot possessed high leaves number (3.583 ± 0.515, 3.833 ± 0.389) and greatest root initiation. However, moderate shoot length (5.050 ± 0.585), leaves number (3.750 ± 0.965) and high root initiation were obtained by 100% TSW. Both lower IAA conc. (0.5 ppm) and higher conc. (2.5 ppm) have less influence on shoot length and leaves number than other treatments. All morphological parameters of tomato explants were stimulated by moderate IAA + BA combination, (1.8 + 2.5 ppm). Similar results were observed by *C. vulgaris* extract when cultivated on BG11 supplied with 0.5 IAA + 10 BA ppm. In addition, moderate shoot length, leaves number and root initiation were reported by applying the crude extract of *C. vulgaris* grown on 100% TSW, Table 8 and Fig. 2.

**Table 8:** The effect of *Chlorella vulgaris* crude extracts (Grown on BG11 and 100% TSW) with optimum conc. of acetic acid (IAA) and benzyl adenine (BA) (obtained from dose response curve) on Tomato plant

Parameter	Shoot Length (cm)	Leaves, numbers	Root initiation	Branching	Leaf expansion
Control (MS medium)	5.217 ± 0.469^bcd^	3.500 ± 0.674 ^ab^	+ + +	-	-
DMSO	5.083 ± 0.925 ^c d^	3.417 ± 0.669^ab^	+ + +	-	-
IAA ( 0.5 ppm)	4.850 ± 0.454^cde^	3.083 ± 0.900^bc^	+ + + + + +	-	-
IAA (1.8 ppm)	6.850 ± 1.588^a^	3.583 ± 0.515^ab^	+ + + + + +	-	-
IAA (2.5 ppm)	6.008 ± 0.734 ^ab^	3.750 ± 0.452^a b^	+ + +	-	-
BA (10 ppm)	4.533 ± 0.624^c de^	2.833 ± 0.577^c d^	+	-	+
BA (25 ppm)	4.442 ± 0.516^c d e^	3.500 ± 0.674^a b c^	+	+ +	+
BA (50 ppm)	4.425 ± 0.636^de^	3.333 ± 0.651^a b c^	-	+ + +	+ + +
IAA + BA (0.5 + 10) ppm	4.008 ± 0.478^e^	3.167 ± 0.577^abc^	+ + +	-	-
IAA + BA (1.8 + 25) ppm	4.533 ± 0.414^de^	3.667 ± 0.492^ab^	+ + +	+	-
IAA + BA (2.5 + 50) ppm	4.525 ± 0.398 ^de^	2.500 ± 0.674^c^	+	-	-
*C. vulgaris* IAA optim. conc.(BG-11)	5.650 ± 0.678^b c^	2.917 ± 0.996 ^bc^	+ + + + + +	-	-
*C. vulgaris* BA optim. conc. (BG-11)	4.808 ± 0.543^b cd^	3.000 ± 0.603^bcd^	+ + + +	-	-
*C. vulgaris*(100%TSW) IAA optim. conc	5.050 ± 0.585 cd	3.750 ± 0.965^a b^	+ + + + +	-	-
*C. vulgaris* (100%TSW) BA optim. conc	5.5750 ± 0.3306^a^	2.917 ± 0.515^c d^	+ +	-	-

*TSW* treated sewage wastewater (-): Absence (6 +): the highest (5 +):high (4 +):moderate (3 +):low ( +):The lowest *DMSO* Dimethyl sulfoxide, *MS* Murashige and Skoog medium

The values are the mean of three replicates ± SD

Values followed by different letters are significantly different (p ≤ 0.05)

### Soybean experiment

**Fig 3:**
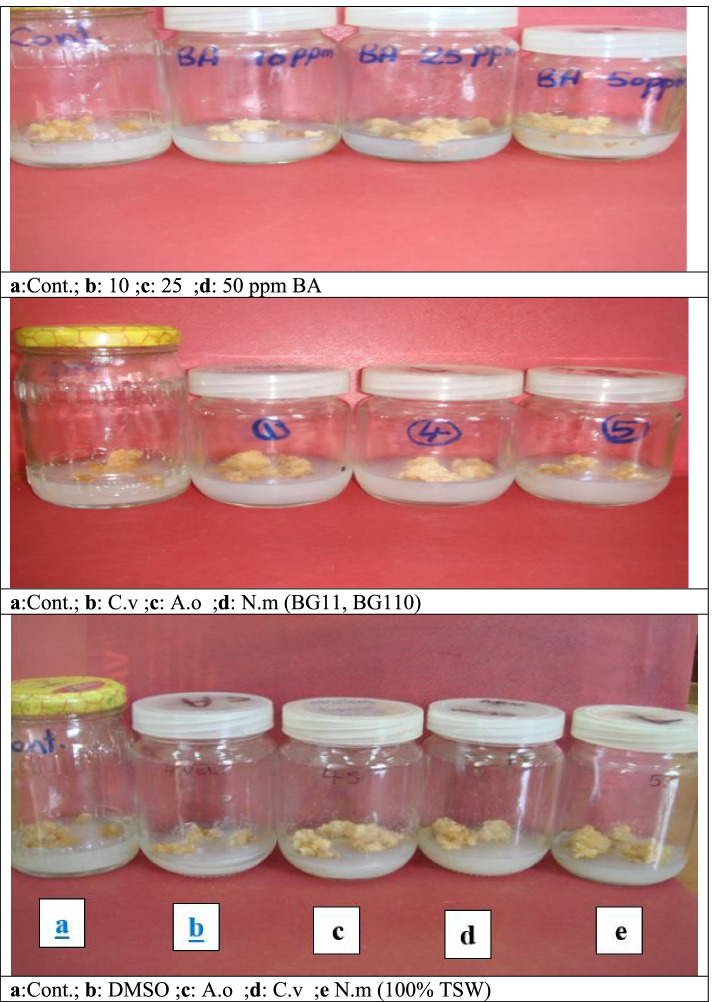
Effect of synthetic BA different concentrations as well as algal crude extracts (grown on BG11 and TSW media) on soybean callus fresh weight (g).

Table 9 and Fig. 3 showed the application of cytokinin (BA) bioassay experiment using different concentrations. as well as the effect of crude extracts of the cyanobacterial species *A. oryzae* and *N. muscorum* grown on BG11_0_ and 100% TSW media and the green alga *Chlorella vulgaris* which was grown on BG11 and 100 TSW % on fresh weight of soybean callus. Basal MS medium represent the control.

**Table 9:** Effect of crude extracts of algal species on soybean callus fresh weight ( as gm)

Samples Conc	Initial weight (gm)	Final weight (after 28 days) (gm)	Net callus weight (gm)
Callus Wt			
Control (MS medium)	1.9250 ± 0.115	1.8320 ± 0.104	-0.0930
BA 10 ppm	1.657 ± 0.197	2.8213 ± 0.165	1.164
BA 25 ppm	2.0600 ± 0.073	4.8997 ± 0.079	2.840
BA 50 ppm	1.2590 ± 0.088	3.0413 ± 0.021	1.782
*A.* o*ryzae* ext. ( BG-110)	1.7637 ± 0.170	3.9357 ± 0.049	2.172
*A. oryzae* ext. (100% TSW)	2.604 ± 0.186	3.214 ± 0.461	0.610
*C. vulgaris* ext. ( BG11)	1.1230 ± 0.023	2.839 ± 0.601	1.716
*C. vulgaris* ext. (100% TSW)	2.0093 ± 0.019	2.605 ± 0.473	0.596
*N. muscorum* ext. ( BG-110)	1.6507 ± 0.053	3.9357 ± 0.049	2.285
*N. muscorum ext.* (100% TSW)	2.524 ± 0.230	3.214 ± 0.461	0.690

The values are means of data from 3 replicates ± SD. Values followed by different letters are significantly different (*p* < 0.05)

*MS* Murashige and Skoog medium, *BA* Benzyl adenine, *TSW* Treated sewage wastewater

The callus weight in control plant was highly decreased (-0.093 g) while the addition of BA in different concentrations. (10, 25 and 50 ppm) induced significant increase in callus weight which reached its maximum at BA concentration 25 ppm (2.8397 g).The obtained results concerning soybean fresh weight, revealed that *A. oryzae* and *N. muscorum* extracts (contained BA) after growth on BG11_0_ medium (contained 10–25 ppm BA) induced 1.5-fold increase in callus fresh weight, while when growth on 100% TSW medium (contained > 10 ppm BA) was showed to be less effective in fresh weight increment. Concerning, *C. vulgaris* the results showed that, its extract (contained BA) after growth on BG11 (contained 10–25 ppm BA) induced a moderate effect, but when growth on 100% TSW medium (contained > 10 ppm BA) it was showed to be less effective in fresh weight increment.

The analysis of aqueous commercial seaweed filtrate, micro-algal or cyanobacterial extracts in many published researches revealed that, they contained not only different plant growth hormones (auxins, cytokinin’s, and gibberellins), but also polyamines, betaine, brasinosteroids, in addition to micro-, macronutrients and vitamins to which attributed the growth improvement [29–32]. So, they were termed bio-fertilizers or bio-stimulants and widely used as nontoxic, biodegradable and cheap fertilizers to replace the toxic expensive chemical ones [5, 33].

Plant growth regulators are universally used for enhancing growth and development of many economic crops and vegetables [9, 11, 31, 32, 34]. They are naturally occurring organic compounds which influence the physiological processes that control cell division, embryogenesis, as well as the sequential growth and differentiation processes that are involved in the course of plant reproduction [31, 35, 36]. The endogenously produced plant hormones (especially IAA) in algal and cyanobacterial filtrates or extracts were correlated with root promotion, cell elongation, division, tissue differentiation and responses to light and gravity [37, 38]. Cytokinin’s are produced by many algae and cyanobacteria and play a vital role in regulating growth and morphogenesis. It induces shoots [39], direct and indirect plant regeneration [40], induce callus irrespective of explants used [9] as well as plant regeneration from callus by changing the applied percentage of IAA and cytokinin (the key hormones).

The obtained results in the application experiment were in agreement with the results obtained by Suresh et al., [34] who treated crop plants (*zea mays,sorghum bicolour*) with heterocystous cyanobacterial filtrate (0.2–0.5%) which promoted grain seedling growth. Also, using similar bio-stimulants [35, 41] reported an amplification of seed germination percentage and root, shoot lengthening of the germinated plants. Moreover, micro-algal extracts (*Chlorella vulgaris, Scenedesmus obliquus, S. quadricauda and Cladophoropsis gerloffi*) improved lettuce seedlings quality and quantity [6, 33], sugar beet early stages of growth [36] and tomato [31, 32, 42]. Meanwhile, seaweed extracts and seaweed commercial filtrates were widely applied on canola plant by Hashem et al*.*, [5], where it improved growth, yield and alleviated the harmful effect of salinity stress. It was also applied to *Ocimum santicum* [43] to *Brassica chinensis* [8], to water stressed tomato [32] and to crop plants [30]. The stimulatory effect of seaweed filtrates and microalgal extracts were proved to be due to the presence of many growth regulators as auxins, cytokinins, gibberellin, polyamines and betaine in addition to different macro-and micro-nutrients as reported by many investigators [44–49].

## Conclusion

We have successfully detected and determined auxins and cytokinin (IAA and BA) which were endogenous produced phytohormones by cyanobacteria (*A. oryzae* and *N. muscorum*) and Chlrophyta (*C. vulgaris*) species. The extracts of algae grown on different conc. of treated wastewater combined with BG11 or BG11_0_ media showed a significant effect on morphological parameters of tomato plantlets (shoot length, branching, leaves number, and root initiation) and fresh weight of soybean callus bioassays. Algal and cyanobacteria extracts contained hormones, vitamins, enzymes, many micro and macro nutrients which may be used as biostimulants (or biofertilizers) for different plant growth and development.

## Data Availability

The data used and analyzed in this study are available from the corresponding author on reasonable request.
